# Evaluation of the prematurity retinopathy and other eye changes in the newborn

**DOI:** 10.31744/einstein_journal/2022AO6692

**Published:** 2022-05-02

**Authors:** Thiago Gonçalves dos Santos Martins, Leticia de Araújo Franco Andreghetto, Rafael Maciel Brito, Luciane Benitez Provenzano, Susan Fowler

**Affiliations:** 1 Hospital Municipal e Maternidade Escola Dr. Mário de Moraes Altenfelder Silva São Paulo SP Brazil Hospital Municipal e Maternidade Escola Dr. Mário de Moraes Altenfelder Silva, São Paulo, SP, Brazil.; 2 Walden University Minneapolis MN United States Walden University, Minneapolis, MN, United States.

**Keywords:** Ophthalmology, Infant, newborn, Risk factors, Retinopathy of prematurity

## Abstract

**Objective:**

To assess the prevalence of ophthalmologic manifestations in newborns in a maternity hospital in the city of São Paulo, SP, and the main risk factors related with the development of retinopathy of prematurity.

**Methods:**

A retrospective, longitudinal study with patients born from 2015 to 2017 who required ophthalmological evaluation. The research variables were obtained by analysis of the newborn medical charts.

**Results:**

A total of 773 patients were studied. The sample consisted of 288 examinations performed by indication of gestational age ≤32 weeks: 118 (42.4%) in 2015, 105 (42.2%) in 2016, 65 (26.4%) cases in 2017. There were 329 evaluations indicated due to birth weight: 113 (40.6%) in 2015, 108 (43.4%) in 2016, and 108 (43.9%) in 2017. The prevalence of associated risk factors was 97 (34.9%) cases in 2015, 96 (38.6%) in 2016, and 54 (22%) in 2017, followed by mechanical ventilation with 82 (29.5%) cases in 2015, 64 (25.7%) in 2016 and 41 (16.7%) in 2017, and continuous positive airway pressure with 59 (21.2%) cases in 2015, 72 (28.9%) in 2016, and 46 (18.7%) in 2017. For the other indications, the evaluations performed due to congenital syphilis were the majority in the 3-year period of the study, with 55 (19.8%) newborns in 2015, 54 (21.7%) in 2016, and 59 (24.0%) in 2017. The most prevalent ophthalmologic diagnosis was retinopathy of prematurity, with 79 (35.3%) cases in 2015, 64 (32.2%) in 2016, and 41 (24.1%) in 2017.

**Conclusion:**

Most neonates born in the organization do not present risk factors for ophthalmological manifestations. Retinopathy of prematurity was the disease with greater strength of association found in our study. For the other indications, the evaluations performed due to congenital syphilis prevail in the 3- year period of the study.

## INTRODUCTION

Vision is one of the most important basic senses, and the first 18 months of life are the most critical period of its development.^(1-3)^ According to the World Health Organization (WHO), there are approximately 1.4 million children with vision disabilities in the world. Approximately 80% of causes of blindness in children are preventable or treatable.^(3-5)^ In Brazil, studies carried out in schools for students with visual impairment and in low vision services point to chorioretinitis due to toxoplasmosis and childhood cataract, as the main causes, and congenital glaucoma and retinopathy of prematurity (ROP) as the most prevalent ones.^(2)^

After birth, active visual screening facilitates the detection of potential causes of treatable eye anomalies.^(3,4)^ All neonates must undergo the red reflex test before discharge from the maternity hospital and be followed-up for at least two to three times a year, for the first three years of life.^(2)^ In case of altered red reflex, the patient should be referred for specialized ophthalmologic evaluation.^(3,4)^

Retinopathy of prematurity is among the leading causes of child blindness.^(4,5)^ The American Academy of Pediatrics (AAP), American Association of Certified Orthoptists (AACO), American Association of Pediatric Ophthalmology and Strabismus (AAPOS) and American Academy of Ophthalmology (AAO) recommend screening infants born at 28 weeks’ gestational age (GA) or less, or with a birth weight of 1,500g or less.^(6,7)^ According to the Brazilian Council of Ophthalmology (CBO) and the Brazilian Society of Pediatrics (SBP), ROP screening is indicated in premature infants born with a birth weight <1,500g or GA ≤32 weeks.^(2)^ This recommendation, with a higher confidence interval (CI), aims to select a larger number of newborns, therefore reducing childhood blindness cases due to lack of adequate neonatal screening.^(2,8-22)^

In addition to GA and birth weight, there are other indications for a newborn to have an ophthalmological evaluation in the neonatal period. According to the protocol used at the *Hospital Municipal e Maternidade-Escola Dr. Mário de Moraes Altenfelder Silva* (HMEC), congenital infections (toxoplasmosis, rubella, cytomegalovirus, and syphilis), altered red reflex test, and genetic syndromes are considered indications for eye fundus examination.

## OBJECTIVE

To evaluate the prevalence of ophthalmologic manifestations in newborns in a maternity hospital in the city of São Paulo, SP, and the main risk factors related with the development of retinopathy of prematurity.

## METHODS

### Design

This study used a cross-sectional design with a retrospective analysis of medical records of newborn infants who required ophthalmological evaluation at the HMEC maternity hospital in São Paulo, from 2015 to 2017.

We compared the evolution of all variables between 2015 and 2017. Therefore, the test for the equality of two proportions was used, and the relative frequencies (rates) were always calculated for the total number of participants in each year.

From the data obtained between January 1^st^, 2015 and December 31^st^, 2017, we compared the total rate of ophthalmic evaluations required for each year, based on the total number of live births in each year of the study period. We evaluated a total of 773 newborns who required ophthalmological examination in the neonatal period during hospitalization at the HMEC, distributed among the three-year period studied.

### Eligibility criteria

Patients born from January 1^st^, 2015 to December 31^st^, 2017, with indication for ophthalmologic evaluation in the neonatal period were included. To minimize methodological bias, medical records with incomplete data in the ophthalmological evaluation form were excluded from the study.

### Data analysis

The results were obtained through direct analysis of medical records. For statistical analysis, the following tests were used: the test for the equality of two proportions and the χ^2^ test. The p-value was 0.05 (5%), and the confidence interval was 95%. The data were analyzed by a researcher and checked by another researcher.

### Ethical considerations

At all stages of this research, the ethical aspects for research involving human beings were respected, in accordance with Resolution 466/2012 of the National Health Council. Data collection for this research was carried out after approval by the HMEC Research Ethics Committee # 3.020.015 CAAE: 00487018.5.0000.5454), and the Informed Consent Form was waived.

## RESULTS

The distribution per sex in the years studied did not show any statistical difference. Male newborns accounted for 50.4% in 2015, 51% in 2016, and 50.8% in 2017.

In the period analyzed, the sample consisted of 288 examinations performed for the indication of GA ≤32 weeks, distributed as follows: 118 (42.4%) of a total of 278 in 2015, 105 (42.2%) out of 249 in 2016, with a significant drop in 2017, with only 65 (26.4%) cases out of 246 cases who were eligible for this study. Compared to other years, there was a statistical difference in 2017, in which the rate of infants born with GA ≤32 weeks was lower (26.4%), and the rate of neonates born with GA ≤32 weeks was the highest in the study period (73.6%) (Table 1).

**Table 1:** Ophthalmologic evaluations per gestational age during the period of study

Gestational age	2015	2016	2017
≤32 weeks	118	105	65
>32 weeks	160	144	181

Results expressed as n.

During the period, 329 ophthalmological evaluations were carried out for the indication of low birth weight, and distributed as follows: 113 (40.6%) cases out of a total of 278 in 2015, 108 (43.4%) cases out of 249 in 2016, and 108 (43.9%) cases out of 246 in 2017. There was no statistical difference among the years in the rate of indications for low birth weight.

During the study period, all medical records were analyzed in search of other indications for ophthalmological evaluations, such as congenital infection, altered red reflex test, and genetic syndromes. Table 2 shows the number and percentage of newborn infants who met this criterion.

**Table 2:** Other indications for ophthalmological evaluation of newborns

Causes of ophthalmic evaluation	2015	2016	2017
Toxoplasmosis	41 (14.7)	35 (14.1)	28 (11.4)
Rubella	6 (2.2)	6 (2.4)	4 (1.6)
Cytomegalovirus	31 (11.2)	12 (4.8)	11 (4.5)
Syphilis	55 (19.8)	54 (21.7)	59 (24.0)
Red reflex test	23 (8.3)	28 (11.2)	23 (9.3)
Genetic syndrome	6 (2.2)	6 (2.4)	3 (1.2)
Total	162	141	128

Results expressed as n (%).

The prevalence of risk factors associated with the development of ROP was evaluated, including invasive mechanical ventilation, continuous positive airway pressure (CPAP), use of oxygen, suspected or confirmed sepsis, central nervous system infection, use of phototherapy, and presence of intracranial hemorrhage (Table 3).

**Table 3:** Risk factors during the period studied

Risk factors	2015	2016	2017
Mechanical ventilation	82 (29.5)	64 (25.7)	41 (16.7)
CPAP	59 (21.2)	72 (28.9)	46 (18.7)
Oxygen	97 (34.9)	96 (38.6)	54 (22.0)
Sepsis	26 (9.4)	17 (6.8)	26 (10.6)
CNS infection	10 (3.6)	7 (2.8)	3 (1.2)
Phototherapy	90 (32.4)	85 (34.1)	70 (28.5)
ICH	64 (23.0)	39 (15.7)	13 (5.3)
Total	428	380	253
p values of risk factors	2015	2016	
Mechanical ventilation			
2016	0.331		
2017	<0.001	0.014	
CPAP			
2016	0.041		
2017	0.471	0.008	
Oxygen			
2016	0.384		
2017	0.001	<0.001	
Sepsis			
2016	0.290		
2017	0.642	0.139	
CNS infection			
2016	0.610		
2017	0.081	0.208	
Phototherapy			
2016	0.668		
2017	0.331	0.173	
ICH			
2016	0.033		
2017	<0.001	<0.001	

Results expressed as n (%).

CPAP: continuous positive airway pressure; CNS: central nervous system; ICH: intracranial hemorrhage.

Most ophthalmological evaluations performed had no change, with 142 (63.4%) cases in 2015, 129 (64.8%) cases in 2016, and 128 (74.7%) cases in 2017. The most prevalent diagnosis was ROP, with 79 (35.3%) cases in 2015, 64 (32.2%) cases in 2016, and 41 (24.1%) cases in 2017. Most cases analyzed required no surgical or laser treatment.

We analyzed how many patients required surgery or laser treatment among the patients diagnosed as ROP (Table 4).

**Table 4:** Ophthalmological treatment of patients diagnosed with retinopathy of prematurity

Eye treatment	2015	2016	2017
Follow-up	75 (33.5)	63 (31.7)	35 (20.6)
Discharge	144 (64.3)	128 (64.3)	128 (75.3)
Surgery	0	0	5 (2.9)
Referral to other organizations	0	5 (2.5)	0
Laser	5 (2.2)	2 (1.0)	1 (0.6)
Total	224	198	169

Results expressed as n (%).

The distribution of newborns undergoing eye examination was compared with the rate of examinations requested and not performed in the same period. These were newborns who were referred for a fundus examination after hospital discharge, on an outpatient basis, and, for some reason, did not return. The rate of exams not performed was 19.4% in 2015, 20.1% in 2016, and 30.9% in 2017. The increase in number of eye exams not performed in 2017 was significantly different from 2015 (p=0.002) and 2016 (p=0.006).

## DISCUSSION

The causes of childhood blindness vary according to a country’s level of socioeconomic development, and approximately 80% of them are preventable or treatable. Childhood blindness has a great economic impact on society, due to the high life expectancy of the affected infants. Adequate training of neonatologists and all healthcare workers responsible for screening for these conditions, such as using the red reflex test, is of paramount importance, since sensitivity and specificity of this test are directly related to the experience of the examiner.

Despite the high rate of live births registered at the site and during the study period, the number of newborns evaluated by an ophthalmologist is small, supporting the hypothesis that a higher rate of neonates in the organization do not present risk factors for ophthalmologic manifestations.

In the period analyzed, the sample consisted of 288 examinations performed for the indication of GA ≤32 weeks, and the number of examinations was gradually reduced over the years, which may be related to an improvement in prenatal care over time, leading to a lower premature birth rate. Comparing the years, there was no significant difference in birth weight rates, and birth weight >1,500g was more prevalent in all years.

As to other indications, most evaluations were performed for congenital syphilis in the three-year period of the study, followed by congenital toxoplasmosis and altered red reflex test. In Brazil, during the last 5 years, there was a steady increase in the number of syphilis cases in pregnant women, which could be attributed, in part, to increased testing coverage, a rise in use of rapid tests, a drop in use of condoms, and a worldwide penicillin shortage. In addition, the improvement of the surveillance system may be reflected in the increase of reported cases.^(23,24)^

In the city of São Paulo, there was an increase in reported cases and in the incidence rate of congenital syphilis between 2015 and 2017, coinciding with the period studied. These data are consistent with the international literature, which has also intensified studies on the impact of this risk factor on eye diseases.^(23,24)^ In this study, no eye lesions were found in patients with suspected or confirmed congenital syphilis.

In a similar assessment carried out in Pernambuco, between April and October 2000, 3,280 newborns were examined, and the abnormalities found in the eye examination included 43 cases of infectious conjunctivitis (3.0%), 5 cases of congenital cataract (0.4%), and 28 cases of subconjunctival hemorrhage (2.0%). Blepharophimosis was observed in two patients (0.3%). The red reflex test was altered in 24 cases (3.4%). On fundus examination, retinal hemorrhage in the posterior pole was the most common alteration found, occurring in 255 newborns (7.8%), and macular involvement was identified in 11 (4.3%) cases.^(25)^

Retinopathy of prematurity had two well-identified epidemic phases: in the 1950s, attributed to the extensive use of oxygen in neonatal intensive care units (NICU) and, in the 1970s, due to the increased survival of premature infants born with extremely low birth weight.^(26-30)^ The progression of ROP severity may be related to multiple factors associated with treatment and care at NICU, mainly the use of oxygen, associated or not with mechanical ventilation.^(27-30)^ A careful monitoring of supplemental oxygen administration, management of the patient’s oxygen saturation, and the maintenance of residual respiratory function using CPAP adjustments and non-invasive ventilation techniques are related to less severe forms of the disease.^(2,27-30)^

Invasive mechanical ventilation, CPAP and oxygen use had great statistical significance, showing a gradual decrease over the years analyzed. Leading the prevalence among the associated risk factors were the use of oxygen and CPAP, which are the main factors related to the development of retinopathy of prematurity described in the literature.^(19)^ In addition to the use of oxygen, other risk factors for ROP have been mentioned in the literature, such as neonatal sepsis, congenital infections, blood transfusions, intracranial hemorrhage, asphyxia, and vitamin E deficiency.^(19)^ The number of ROP cases analyzed has decreased over the years, and this scenario may be the result of better prenatal care.

In a cross-sectional observational study carried out in Brazil, at a NICU in the state of Rio Grande do Sul, involving 139 newborns between 2002 and 2004, the prevalence of ROP was 27.2%, affecting 31 neonates born with birth weight ≤1,500g and/or GA ≤32 weeks.^(5)^ In the same study, in 72.8% of cases evaluated, no eye abnormalities were found, similar to the results of this study, in which 63.4% of all eye examinations performed in 2015 were considered normal, 64.8% in 2016, and 74.1% in 2017.^(29)^

In 1991, Charles et al. studied the incidence and characteristics of ROP in a low-income urban population in the United States and reported a prevalence of ROP in 72% of infants born with birth weight <1,200g, and in 66% of neonates born with GA ≤32 weeks.^(30)^

Larsson et al. studied 392 newborn infants in Stockholm, Sweden, and published, in 2002, a prevalence of 25.5% of ROP.^(21)^ Literature data show the occurrence of ROP is mainly associated with low GA and birth weight.^(2)^

Patients with diseases in more severe stages or who required specialized follow-up were referred to reference centers in the city of São Paulo, due to the organization limitations in monitoring or solving cases.

When analyzing the results, it was observed that the evolution of the number of examinations not performed showed a significant increase from 2015 to 2017. This reinforces the need to adequately inform parents of the importance of ophthalmological monitoring of newborns, and to develop public policies that facilitate the return of these patients to the maternity hospital, such as the provision of transportation assistance, since the majority of the population studied had a low socioeconomic level.

### Study limitations and future perspectives

The data reflect the prevalence of ophthalmological manifestations in patients seen at a hospital in Brazil, which cannot be generalized to different contexts. A study with a cross-sectional design with retrospective analysis depends on database records. New studies with a larger number of patients in different countries may help to improve understanding of ophthalmological manifestations in newborns. New studies comparing large databases with the aid of data warehousing can be useful for near real-time data analysis, benefiting patient care and public health strategies. One of the greatest strengths of this type of database is that, in addition to help providing answers to many questions on outcomes, it can also aid in the development of trials and prospective studies requiring targeted data. It is a useful technology for data-intensive organizations that are getting larger and more complex, which data cannot be processed by traditional applications.

The development of artificial intelligence algorithms and portable retinal scanners have helped screening children in areas with poor eye care. Telemonitoring can facilitate the monitoring of these eye diseases. Telemedicine adds to the training of physicians, in addition to avoiding patients having to travel great distances to be treated.

However, the training of these professionals and the awareness of professionals and parents about the importance of eye examination at this age are important, reducing the economic cost caused by eye diseases that can be prevented with proper diagnosis and treatment.

## CONCLUSION

The majority of newborns analyzed had no risk factors for ophthalmologic manifestations. Most of the eye examinations performed did not show any abnormalities. Retinopathy of prematurity was the most prevalent eye disorder during the study period, followed by ophthalmological evaluations of newborns of mothers infected with syphilis during pregnancy. Invasive mechanical ventilation, continuous positive airway pressure and oxygen use were among the main risk factors correlated with the development of retinopathy of prematurity, and their use was reduced over the years studied.
